# A novel psychrophilic alkaline phosphatase from the metagenome of tidal flat sediments

**DOI:** 10.1186/s12896-015-0115-2

**Published:** 2015-01-31

**Authors:** Dae-Hee Lee, Su-Lim Choi, Eugene Rha, Soo Jin Kim, Soo-Jin Yeom, Jae-Hee Moon, Seung-Goo Lee

**Affiliations:** Synthetic Biology and Bioengineering Research Center, Korea Research Institute of Bioscience and Biotechnology (KRIBB), Daejeon, Korea; Biosystems and Bioengineering Program, Korea University of Science and Technology (UST), Daejeon, Korea; Present address: Su-Lim Choi, Amicogen, Inc., Jinju, Korea

**Keywords:** Alkaline phosphatase, Metagenome, PhoX, *Escherichia coli*

## Abstract

**Background:**

Alkaline phosphatase (AP) catalyzes the hydrolytic cleavage of phosphate monoesters under alkaline conditions and plays important roles in microbial ecology and molecular biology applications. Here, we report on the first isolation and biochemical characterization of a thermolabile AP from a metagenome.

**Results:**

The gene encoding a novel AP was isolated from a metagenomic library constructed with ocean-tidal flat sediments from the west coast of Korea. The metagenome-derived AP (mAP) gene composed of 1,824 nucleotides encodes a polypeptide with a calculated molecular mass of 64 kDa. The deduced amino acid sequence of mAP showed a high degree of similarity to other members of the AP family. Phylogenetic analysis revealed that the mAP is shown to be a member of a recently identified family of PhoX that is distinct from the well-studied classical PhoA family. When the open reading frame encoding mAP was cloned and expressed in recombinant *Escherichia coli*, the mature mAP was secreted to the periplasm and lacks an 81-amino-acid N-terminal Tat signal peptide. Mature mAP was purified to homogeneity as a monomeric enzyme with a molecular mass of 56 kDa. The purified mAP displayed typical features of a psychrophilic enzyme: high catalytic activity at low temperature and a remarkable thermal instability. The optimal temperature for the enzymatic activity of mAP was 37°C and complete thermal inactivation of the enzyme was observed at 65°C within 15 min. mAP was activated by Ca^2+^ and exhibited maximal activity at pH 9.0. Except for phytic acid and glucose 1-phosphate, mAP showed phosphatase activity against various phosphorylated substrates indicating that it had low substrate specificity. In addition, the mAP was able to remove terminal phosphates from cohesive and blunt ends of linearized plasmid DNA, exhibiting comparable efficiency to commercially available APs that have been used in molecular biology.

**Conclusions:**

The presented mAP enzyme is the first thermolabile AP found in cold-adapted marine metagenomes and may be useful for efficient dephosphorylation of linearized DNA.

## Background

Alkaline phosphatase (AP; EC 3.1.3.1) is a ubiquitous enzyme widely distributed from microorganisms to humans [1] and functions to catalyze the hydrolytic cleavage of phosphate monoesters under alkaline conditions, releasing inorganic phosphate from many phosphate-containing compounds. This enzyme plays important roles in microbial ecology through its involvement in phosphate metabolism [2], signal transduction [3], and bacterial virulence [4]. Among bacterial APs, *Escherichia coli phoA*, which encodes an AP (termed ECAP), has been most extensively studied. ECAP is a homodimeric enzyme requiring two Zn^2+^ ions and one Mg^2+^ per monomer [5], and its active site has a conserved serine residue that is phosphorylated through catalysis [6]. PhoA-like APs are found in many other bacteria and have conserved domains with those in ECAP [5]. Recently, the novel AP genes *phoX* and *phoK* were identified in *Vibrio cholerae* [7] and *Sphingomonas* sp. strain BSAR-1 [8], respectively; these genes were shown to be activated by Ca^2+^ and share no homology with *phoA*.

AP is also a key enzyme in many molecular biology techniques, such as enzyme-linked immunosorbent assays [9], western blots, and immunodetection assays [10]. The favorable properties of AP for these applications are high catalytic activity and thermal instability, which allows simple inactivation by heat treatment [11]. Thus, several thermolabile APs have been isolated and characterized from psychrophilic microorganisms such as the Antarctic strain TAB5 [12], the marine bacterium *Vibrio* G15-21 [13], and the bacterium *Shewanella* sp. SIB1 [14]. Furthermore, the AP enzyme from the marine psychrophile *Sphingomonas* sp. strain BSAR-1 (SPAP) was found to be very efficient in bioprecipitation of uranium as uranyl phosphate under alkaline conditions [8].

Metagenomes have been successfully used for the discovery of novel biocatalysts and biomolecules for biotechnological and pharmaceutical applications [15,16]. To date, no thermolabile APs from metagenomes have been isolated or characterized. This might be due to the limited availability of sensitive and reliable screening systems, which enable the screening of large libraries of metagenome clones [17,18]. Recently, we developed a genetic circuit termed the Genetic Enzyme Screening System (GESS) that allows for the measurement of enzyme activity as *in vivo* fluorescence intensity and is highly useful for high-throughput metagenomic screening of enzymes [19]. The GESS is able to detect the activities of phenol-releasing enzymes, such as esterases/lipases, lyases, cellulases, and phosphatases, which have many applications in biotechnology [19].

Here, we report the first isolation and characterization of a novel thermolabile AP from metagenomes using the GESS. A metagenomic library was constructed with ocean-tidal flat sediments because they were collected from dynamic physicochemical environments with remarkable microbial diversity. We repeatedly screened approximately 10^6^ metagenomic fosmid clones and identified one positive clone that showed significant AP activity. The metagenome-derived AP (mAP) was expressed as a soluble form with a high yield in the periplasmic space of *E. coli*, purified to homogeneity, characterized biochemically, and then applied to the dephosphorylation of linearized plasmid DNA.

## Results

### Rationale of the GESS method

We previously developed a genetic circuit, GESS, for functional screening of phenol-generating enzymes from genomes or metagenomes [19]. DmpR from *Pseudomonas* sp. strain CF600 acts as a transcriptional activator that can be stimulated upon binding to its effector. It regulates the expression of the divergently transcribed *dmp* operons, which are involved in phenol degradation, from the P_o_ promoter in response to aromatic effector compounds (e.g., phenol and cresol) [20]. We took advantage of this regulatory system by placing the P_o_ promoter upstream of enhanced green fluorescent protein (*egfp*) gene to put EGFP expression under the control of DmpR, generating a GESS for detection of phenol and its derivatives [19]. For functional screening of AP, the DmpR of GESS can be activated upon binding to *p*-nitrophenol (*p*NP), which is released from *p*-nitrophenyl phosphate (*p*NPP) hydrolysis by AP, and then turn on the *egfp* expression. Using this GESS, we conducted a systematic screening that allowed the high throughput *in vivo* detection of AP activity from metagenomes.

### Isolation of an AP from a metagenome library

**Figure 1:**
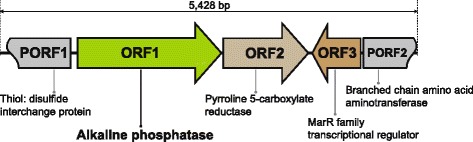
**Identification of putative alkaline phosphatase from metagenome.** Sequencing of subclone (pSTV28-AP6) expressing alkaline phosphatase activity resulted in the assembly of a 5,428-bp contig. Three intact ORFs (ORF1 - ORF3) and two partial ORFs (PORF1 and PORF2) with conserved domains were identified through BLAST search.

A metagenomic library was constructed from DNA isolated from ocean-tidal flat sediments and encompassed approximately 80,000 clones with an average insert size of 30 kb. This library was screened using *p*NPP as a substrate to detect AP activity. Using the strategy described in the Methods section, we discovered six clones exhibiting strong AP activity. Further screening resulted in the identification of a single positive fosmid clone, designated pFOS144C11, which carried a DNA insert of approximately 35 kb. After randomly shearing the DNA, 3 to 7 kb fragments were ligated into pSTV28 and a sublcone designated pSTV28-AP6 was identified based on fluorescence signals and AP activity. The sequence of this clone was determined (Figure 1). Among three intact ORFs in pSTV28-AP6, an ORF of 1,824 nucleotides encoding a putative AP of 607 amino acids, with a deduced molecular mass of 64 kDa, was found through a BLAST search. The deduced amino acid sequence of *mAP* gene was compared with other protein sequences of APs available from the NCBI database using the BLASTP program. mAP exhibited significant homology with the AP superfamily, and the level of sequence identity showed the highest similarity with nucleotide phosphodiesterases from psychrophilic *Sphingopyxis* sp. MC1 (85% amino acid sequence identity, GenBank accession no. WP 003042625) and *Sphingopyxis alaskensis* RB2256 (72% amino acid sequence identity, GenBank accession no. YP_615103). In addition, this mAP was also highly similar to APs from *Sphingobium* sp. SYK-6 (59% amino acid sequence identity, GenBank accession no. YP_004833865) and *Sphingomonas* sp. strain BSAR-1 (58% amino acid sequence identity, GenBank accession no. EF143994) [8].

### Sequence and phylogenetic analyses of mAP

**Figure 2:**
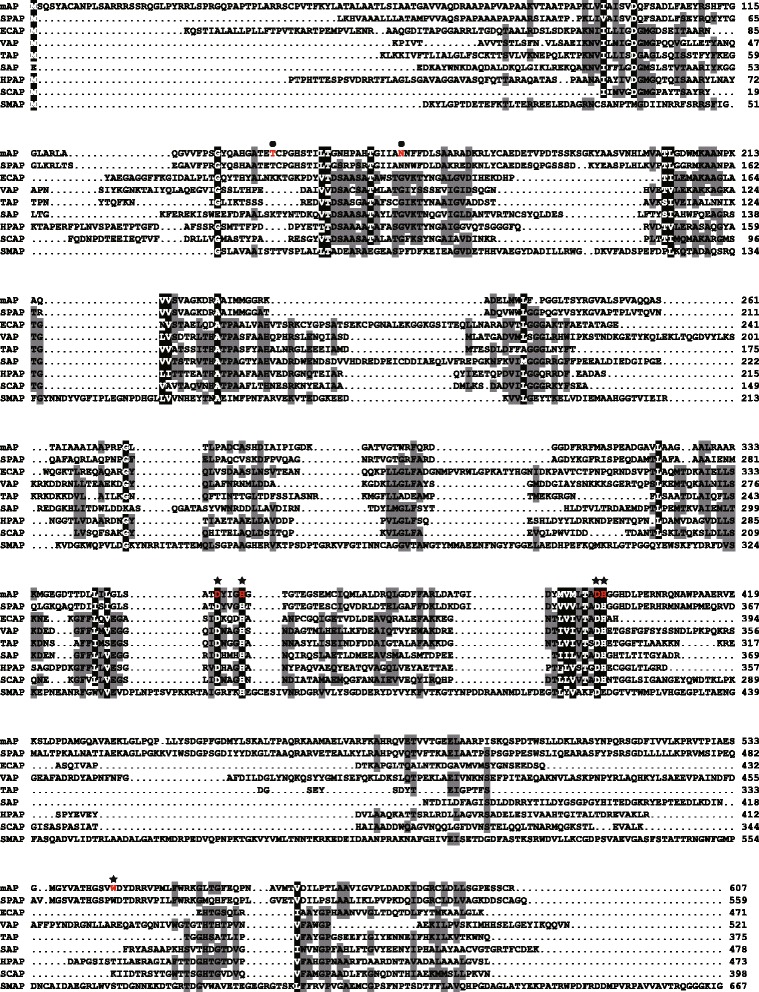
**Multiple alignment of the deduced amino acid sequence of the mAP gene derived from the metagenome with other alkaline phosphatases (APs).** APs are identified by their GenBank or PDB accession numbers: *Sphingomonas* sp. strain BSAR-1 AP (SPAP, ABL96598), *Escherichia coli* AP (ECAP, BAE76164), *Vibrio* sp. G15-21 AP (VAP, AAK94204), Antarctic bacterium TAB5 AP (AAP, CAB82508), shrimp AP (SAP, PDB 1SHQ), human placenta AP (HPAP, PDB 1ZEB), *Shewanella* sp. AP1 AP (SCAP, BAB85685), and *Sinorhizobium meliloti* 1021 AP (SMAP, NP 385195). Highly conserved amino acid residues are highlighted in black, and gaps are denoted by dots. Gray-shaded amino acids are conserved in at least six of the nine APs shown. The amino acid residues forming the metal binding site and catalytic residues of mAP are denoted by asterisks (★) and closed circle (●), respectively. Numbers along the sequences indicate the positions of the amino acid residues starting from the initial Met for each AP.

**Figure 3:**
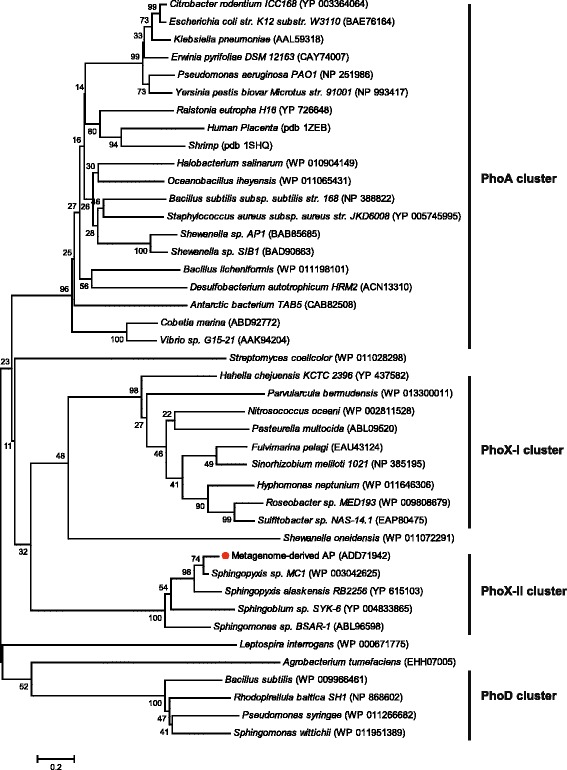
**Phylogenetic analysis of mAP.** A phylogenetic tree based on the similarities of full-length deduced amino acid sequences was constructed with MEGA 5.2 software using the neighbor-joining method. GenBank or PDB accession numbers are given after the species designation. Numbers at nodes are bootstrap values based on 1,000 samplings.

A multiple-sequence alignment analysis of mAP was conducted using APs that showed high similarity with functionally characterized APs (Figure 2). Recently, the Thr89 of SPAP was found to be the catalytic residue [21]; this residue was conserved as Thr138 in mAP. In all other APs, the catalytic residue is a conserved serine (e.g., Ser102 for ECAP); thus, mAP was unique in that its catalytic residue was a threonine (Figure 2). The alignment also identified absolute conservation of six residues (D99, D397, H398, D352, H356, and W545 in mAP) of the active site between SPAP and mAP. The deduced amino acid sequence of mAP was then used for the construction of a phylogenetic tree with full-length amino acid sequences of APs from various organisms (Figure 3). Four distinct clusters were formed: (i) the PhoA cluster, including APs from *E. coli*, shrimp (SAP), and Antarctic bacterial TAB5 (AAP); (ii) the PhoX-I cluster containing most marine bacterial APs and the well-characterized *Sinorhizobium meliloti* AP (SMAP); (iii) the PhoD cluster; and (iv) the PhoX-II cluster, including functionally and structurally characterized SPAP, mAP, and mAP homologs from psychrophilic marine bacteria. The PhoX-I and PhoX-II are well-defined groups in PhoX family, mostly along phylogenetic lines [22]. PhoX-I includes a diverse group of α- and γ-*Proteobacteria* while PhoX-II includes the Gram-positive *Actinobacteria*, most the *Cyanobacteria* and many of the α-*Proteobacteria* encompassing *Sphingopyxis*, *Sphingobium*, and *Sphingomonas*.

### Expression and purification of mAP

**Figure 4:**
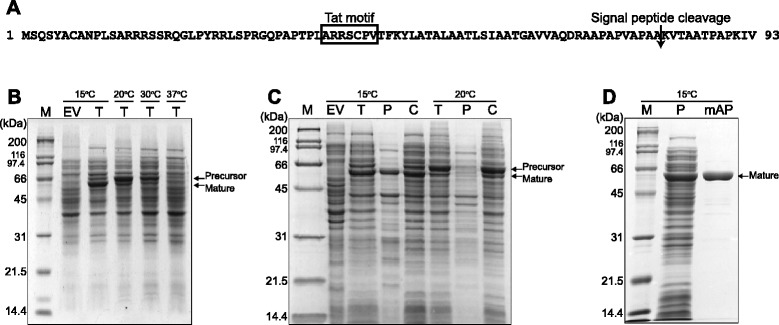
**Signal peptide sequence, heterologous expression, and translocation of mAP. (A)** N-terminal amino acid sequence of mAP showing the predicted Tat motif and signal peptide cleavage site at Ala^81^. **(B)** Expression of mAP in the *E. coli* BL21(DE3) transformant harboring pET-mAP at various temperatures from 15°C to 37°C. Lane M, protein standards; lane EV, total proteins expressed in BL21(DE3) cells with pET-21a at 15°C; lane T, IPTG-induced total proteins of BL21(DE3) with pET-mAP at various temperatures. **(C)** Secretion of mAP. mAP-expressing cells grown with 0.5 mM IPTG for 24 h were fractionated into total (lane T), periplasmic (lane P), and cytoplasmic/membrane (lane C) fractions and subjected to SDS-PAGE. Lanes M and EV are the same as in **(B)**. **(D)** Purification of mAP. mAP expressed at 15°C was purified using the periplasmic fraction. Lane M, protein standards; lane P, IPTG-induced periplasmic fraction; lane mAP; purified mAP protein after dialysis and concentration. Arrows indicate precursor and mature mAP.

To further characterize the mAP enzyme, the 1,824-bp *mAP* was cloned into the pET-21a plasmid and overexpressed in *E. coli* BL21(DE3). When mAP was expressed in *E. coli* cells at 37°C, no protein band with a calculated molecular mass of 64 kDa was detected on sodium dodecyl sulfate-polyacrylamide gel electrophoresis (SDS-PAGE). However, when we expressed *mAP* at various temperatures (15°C, 20°C, or 30°C), mAP expression was increased as the temperature was decreased (Figure 4B). Interestingly, when whole cell extracts from cells expressing mAP were separated by SDS-PAGE, two bands were observed: one band having the calculated molecular mass of 64 kDa, and the other band having a lower molecular mass of approximately 56 kDa (Figure 4B). It is possible that the 56 kDa band represents the secreted and processed form from which the signal peptide cleaved. In order to test this possibility, the localization of mAP was examined when it was expressed at 15°C and 20°C. After cellular fractionation of periplasmic and cytoplasmic proteins using sucrose osmotic shock, SDS-PAGE was conducted. mAP with a molecular mass of 56 kDa was detected in the periplasmic fraction only at 15°C (Figure 4C), indicating that mAP was a cold-adapted AP. The His_6_-tagged mAP enzyme secreted into the periplasm of *E. coli* BL21(DE3) cells was purified using single-step Ni-NTA affinity chromatography (Figure 4D). The purified mAP enzyme exhibited a high specific activity of 1,528 U/mg when *p*NPP was used as the substrate at 37°C. Purified mAP was then used for subsequent biochemical characterization.

### Type II secretion by a putative Tat signal peptide

**Figure 5:**
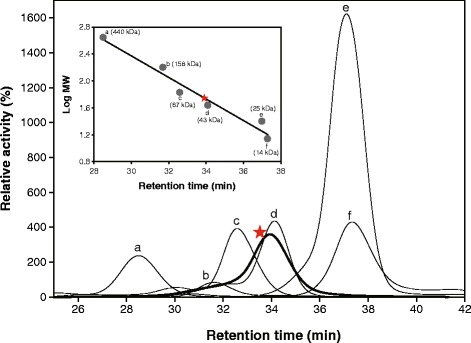
**Molecular mass determination of mAP by size exclusion chromatography.** mAP and six molecular mass standard proteins (peak a, ferritin; peak b, adolase; peak c, albumin; peak d, ovalbumin; peak e, chymotrypsinogen A; and peak f, RNase A) were each subjected to size exclusion chromatography on a Superose 6 HR column that had been pre-equilibrated with 25 mM Tris–HCl buffer containing 150 mM NaCl at pH 7.6. The molecular mass of mAP was estimated by comparing the retention time of mAP with the standard curve (plot of retention time versus logMW) obtained for the molecular mass standard proteins (inset).

Because there was a discrepancy between the calculated (64 kDa) and experimentally determined (56 kDa) molecular mass of mAP, a bioinformatic survey was carried out to predict the signal sequence of mAP. The SignalP 4.1 Server [23] did not identify any significant signal peptide cleavage sites in the deduced amino acid sequence of mAP. However, according to TatP 1.0 Server software [24], which can predict the presence and location of twin-arginine translocation (Tat) signal peptide cleavage sites in bacteria, the first 81 residues of mAP were predicted to be a Tat signal peptide. Indeed, N-terminal amino acid sequencing of the purified 64-kDa and 56-kDa mAPs confirmed the first 81 amino acids as a signal peptide (Figure 4A). The Tat signal peptide, which has the consensus motif SRRxFLK, followed by a membrane-spanning hydrophobic region [25], is a conserved feature among PhoX proteins [22]. Based on these results, we concluded that mature mAP had a length of 527 amino acids and a molecular mass of 56 kDa, in accordance with the expression results of mAP (Figure 4B and C). In order to determine the molecular mass of mAP in its native form, mAP was subjected to size exclusion chromatography. The molecular mass was determined by comparing its elution time with those of standard proteins. One major peak of mAP appeared when the elution time was 33.9 min. Regression analysis to compare the molecular mass of standard proteins and the elution time indicated that the molecular mass of active mAP was around 50 kDa (Figure 5), which suggested that mAP was monomeric in its native form.

### Enzymatic properties of mAP

Next, we investigated the substrate preference of the purified mature mAP using various substrates. mAP demonstrated significant phosphatase activity with *p*NPP and with phosphorylated metabolites including nucleotides and carbohydrates (Table 1). Except for phytic acid and D-glucose 1-phosphate, mAP dephosphorylated all the tested substrates. While mAP showed broad substrate specificity, the highest activity was detected with ADP, followed by dATP, dGTP, and AMP. However, mAP was unable to hydrolyze *bis*-*p*NPP, indicating that it did not have phosphodiesterase activity. The previously reported SMAP also demonstrated significant phosphatase activity with most natural substrates, which cover all major classes of known phosphorylated metabolites [22].

**Table 1:** **Substrate specificity of purified mAP**

**Substrates (1 mM)**	**P**_***i***_ **released relative to that from** ***p*** NPP*
*p*NPP	100
*bis-p*NPP	ND
AMP	39
ADP	76
ATP	25
dATP	44
dCTP	38
dGTP	39
dTTP	37
D-Glucose 1-phosphate	0.2
*O*-Phosphoethanolamine	3
Phosphoenolpyruvate	18
Phytic acid	ND
Paraoxaon	ND
Methyl parathion	ND

*The P_*i*_ released from *p*NPP was set at 100%. ND: not detected. All results are the means of three experiments wherein variation was less than 15%.

**Figure 6:**
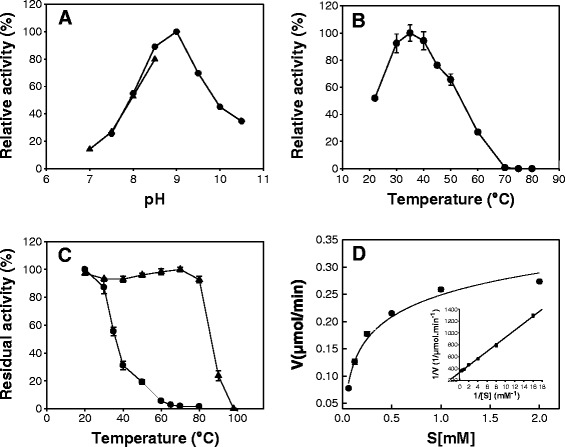
**Effects of pH and temperature on mAP activity.** AP activity was measured by a spectrophotometric method using *p*-nitrophenyl phosphate (*p*NPP). **(A)** Optimum pH. Enzymatic activity was assayed at 37°C in the presence of 0.5 mM *p*NPP as a substrate using 50 mM Tris–HCl buffer (pH 7.0–8.5) or 50 mM DEA buffer (pH 8.0 to 10.5). **(B)** Optimum temperature. Enzymatic activity was assayed at 20–80°C in the presence of 0.5 mM *p*NPP as a substrate using 50 mM DEA buffer (pH 9.0). **(C)** Thermal stability. Residual activity of mAP (●) was determined at 37°C after 15 min incubation at temperature ranges from 20°C to 100°C. Bacterial AP (▲) was also assayed under the same conditions. **(D)** Kinetic studies of mAP. Enzymatic activity was assayed under standard condition with 0.5 mM CaCl_2_ using a range of *p*NPP.

The apparent optimal pH of mAP was 9.0 in 50 mM diethanolamine (DEA) buffer. The enzyme exhibited at least 70% of its optimal activity over a rather narrow pH range, from 8.5 to 9.5 (Figure 6A) and had significant activity over a pH range from 7.5 to 10.5. Furthermore, thermal activation studies demonstrated that the apparent optimum temperature was 37°C, and 25% of the maximum activity was found at 60°C (Figure 6B). Moreover, the enzyme exhibited at least 80% of its optimal activity over a temperature range from 30°C to 40°C, indicating that mAP was a marine-derived psychrophilic enzyme. Thermal stability was determined from enzyme incubation for 15 min over a range of temperatures (20 – 80°C). The psychrophilic mAP showed remarkable thermal instability, resulting in complete loss of its enzymatic activity, by incubation at 65°C for 15 min (Figure 6C).

Divalent cations, such as Zn^2+^ and Mg^2+^, have been identified as essential cofactors for ECAP [26]. The activity of mAP was completely inhibited by 5 mM EDTA, indicating that it was a metalloenzyme (Table 2). To examine the metal specificity of mAP, the enzyme was dialyzed against 20 mM Tris buffer (pH 7.5) containing 5 mM EDTA. The resulting apoenzyme was assayed for phosphatase activity in the presence of various divalent cations (1 mM each; Table 2). Ca^2+^ was the best metal ion for reconstitution of enzyme activity, followed by Co^2+^ and Zn^2+^, suggesting that mAP was strongly dependent on multiple divalent cations. Mg^2+^ and Cu^2+^, however, had no significant effect on the restoration of enzymatic activity (Table 2).

**Table 2:** **Effects of metal ions on the activity of mAP**

**Metal ions (1 mM)**	% AP activity restored*
None	0.11
Ca^2+^	45.6
Co^2+^	27.3
Zn^2+^	12.8
Ni^2+^	1.66
Mn^2+^	1.44
Mg^2+^	0.81
Cu^2+^	0.24

*Results are expressed as the percentage of activity restored when compared with the enzyme that was not dialyzed against 5 mM EDTA (see Methods). All results are the means of three experiments wherein variation was less than 10%.

The kinetic parameters of mAP were estimated using the *p*NPP as substrate (Figure 6D). The K_m_ of mAP is somewhat lower than the K_m_ values reported for AAP, SAP, and calf intestinal AP (CIAP), but higher than that of SMAP which is a well-characterized PhoX. When compared with the K_m_ value of ECAP, it is almost same (Table 3). In addition, mAP exhibited higher turnover number (k_cat_) and catalytic efficiency (k_cat_/K_m_) than those of ECAP, AAP, CIAP, and SMAP (Table 3).

**Table 3:** **Kinetic parameters of alkaline phosphatases**

**Enzyme**	**Temperature (°C)**	**K**_**m**_ **(mM)**	**k**_**cat**_ **(s** ^**−1**^ **)**	**k**_**cat**_ **/K**_**m**_ **(s** ^**−1**^ **M** ^**−1**^ **)**	**Reference**
ECAP	37	0.17	139	8.2 × 10^5^	[13]
AAP	30	0.50	236	4.7 × 10^5^	[27]
SAP	37	0.69	7,176	1.0 × 10^7^	[28]
CIAP	37	0.40	43	1.0 × 10^5^	[29]
SMAP	37	0.085	2.3	2.7 × 10^4^	[22]
mAP	37	0.18	952	5.3 × 10^6^	This study

ECAP, *E. coli* alkaline phosphatase; AAP, Antarctic alkaline phosphatase; SAP, shrimp alkaline phosphatase; CIAP, calf intestinal alkaline phosphatase; SMAP, *Sinorhizobium meliloti* alkaline phosphatase; mAP, metagenome-derived alkaline phosphatase.

### DNA dephosphorylation by purified mAP

AP is widely used in molecular cloning to remove the 5′-terminal phosphate group from DNA fragments linearized by restriction enzymes, preventing religation of the linearized DNA. Thus, we tested the dephosphorylation efficiency (DE (%)) of mAP against cohesive (HindIII- or PstI-linearized pUC19) and blunt (SmaI-linearized pUC19) ends. The DE (%), which represents the ability of AP to prevent recircularization of linear DNA fragments, was estimated by comparing transformation rates after ligation of dephosphorylated and nondephosphorylated pUC19 plasmids [30]. mAP completely dephosphorylated DNA fragments with 3′-protruding cohesive ends generated by HindIII (Table 4). The DEs (%) of mAP were 91.1% and 99.6% for 5′-protruding cohesive ends generated by PstI and blunt ends generated by SmaI, respectively. The DE (%) of mAP was then compared with that of four different commercial APs (Table 4). The DEs (%) of mAP for both 3′-protruding cohesive ends and blunt ends were comparable with those of commercial APs, whereas the DE (%) of mAP for 5′-protruding cohesive ends was slightly lower than the values determined for SAP, CIAP, and APex heat-labile AP (HLAP) (Table 4).

**Table 4:** **Dephosphorylation activity of mAP and commercial APs**

**Restriction enzyme**	**Dephosphorylation efficiency (%)**				
	**mAP**	**AAP**	**SAP**	**CIAP**	**HLAP**
HindIII	100	99.8	99.5	99.9	99.8
PstI	91.1	87.4	98.0	98.2	95.2
SmaI	99.6	99.4	98.9	100	99.7

mAP, metagenome-derived alkaline phosphatase; AAP, Antarctic alkaline phosphatase; SAP, shrimp alkaline phosphatase; CIAP, calf intestinal alkaline phosphatase; HLAP, APex heat-labile alkaline phosphatase. All results are the means of three experiments wherein variation was less than 10%.

## Discussion

Using the GESS and metagenomic library of ocean-tidal flat sediments, we have identified a novel AP, called mAP, as a new member of the PhoX family of APs. To the best of our knowledge, this enzyme is the first thermolabile AP with functional activity isolated from a metagenome.

The mAP presented here clearly shows that it is distinct from the well-studied PhoA proteins;

(i) The sequence identity between mAP and PhoA family (12% – 15%) was very low compared to the identity observed among PhoAs (30% – 50%). Based on a phylogenetic analysis, mAP evolutionarily belongs to the marine bacterial PhoX family, consistent with its isolation from flat tidal sediments. mAP and its homologs from the psychrophilic marine bacteria *Sphingopyxis* sp. MC1, *Sphingopyxis alaskensis* RB2256, *Sphingobium* sp. SYK-6, and *Sphingomonas* sp. strain BSAR-1 formed a phylogenetically distinct group that did not belong to the PhoA family.
(ii) mAP activity is Ca^2+^-dependent whereas PhoA activity requires Mg^2+^ and Zn^2+^. ECAP is probably the most well-characterized PhoA to date. ECAP requires two Zn^2+^ ions to coordinate the phosphomonoester in the active center [5]. Interestingly, SPAP requires Ca^2+^ and Zn^2+^ for activation, while Mg^2+^ has no effect [21]. PhoX has a different catalytic center that requires Ca^2+^ instead of Zn^2+^ for enzyme activity [7]. Similarly, mAP also required Ca^2+^ for catalytic activity, indicating that its requirement for Ca^2+^ instead of Zn^2+^ has probably been a major factor in its selection over PhoA in flat tidal sediments, where Zn^2+^ often occurs at subnanomolar concentrations [31]. A recent study of the marine metagenomic Global Ocean Survey database showed that PhoX is more widely distributed among marine bacteria than PhoA [1].
(iii) mAP is exported via the Tat pathway whereas PhoA translocation is dependent upon Sec pathway [5]. In contrast to the Sec pathway, the Tat secretion system is able to translocate fully folded proteins across bacterial membranes [32]. The Tat signal peptides are generally longer than Sec signal sequences [33]. mAP was found to have N-terminal 81 amino acids as a Tat signal peptide. The Tat signal peptide of mAP was longer than other previously identified PhoX family proteins that are ranging from 26 to 76 amino acids [22]. In addition, mAP contained a Tat motif (ARRSCPV, residues 39–45) that is slightly different from previously reported Tat signal peptides of PhoX family [34,35]. Interestingly, the Tat signal peptide of mAP was working at only low temperature (15°C) among all tested temperatures (15–37°C), suggesting that it has been evolved as a cold-adapted AP in ocean-tidal flat sediments with dynamic temperature changes. Although SPAP is also released to extracellular space, its signal peptide is not described yet.
(iv) mAP exists as a monomeric enzyme while PhoA is active in the dimeric state. Among characterized PhoA family, ECAP and SPAP are present as a homodimer and multimeric protein, respectively.

Recently, the crystal structure of SPAP was reported and revealed that the key catalytic residue in SPAP is threonine, which is conserved in mAP, instead of serine found in all other APs [21]. In addition, the arginine conserved in key active site of PhoAs is deleted in SPAP. Based on these unique structural features, SPAP represents a new class of APs [21]. Although SPAP and mAP are structurally different from other APs, they share several enzymatic properties with typical PhoXs, such as high specific activity, low substrate specificity, and thermolability. In addition, both SPAP and mAP enzymes require Ca^2+^ for activation and mAP is secreted through the Tat pathway, which are similar to those of other PhoX proteins [1].

Psychrophilic enzymes are frequently associated with longer polypeptide chains compared to their mesophilic counterparts [36]; indeed, this was the case for mAP in the present study. Compared to the majority of the APs in the SwissProt Database, monomeric immature mAP was a relatively large protein (64 kDa) composed of 607 amino acids. Indeed, mAP exhibited remarkable thermal instability and was completely inactivated by incubation at 65°C for 15 min, in contrast to BAP, which retained 98% of its original activity at 70°C. In general, remarkable thermal instability, along with higher catalytic efficiencies at low and moderate temperatures, is properties of psychrophilic enzymes [37]. Therefore, an AP from psychrophiles exhibiting such properties could be of significant biotechnological interest.

APs are used in various molecular biology applications for the dephosphorylation of plasmid DNA prior to cloning in order to prevent recircularization, for the dephosphorylation of 5′-nucleic acid termini before 5′-end labeling by polynucleotide kinase, and for the removal of dNTPs and pyrophosphate from polymerase chain reaction assays. However, the AP has to be carefully removed after dephosphorylation to avoid interference with subsequent steps. Furthermore, ECAP and CIAP, which are preferred enzymes for these applications, are heat-stable and require the addition of detergents for inactivation. Thus, thermolabile APs are excellent alternatives as they are inactivated by moderate heat treatment and would therefore allow subsequent steps to be performed in the same test tube while minimizing nucleic acid losses. We examined the DE (%) using a linear cohesive or blunt-ended pUC19 plasmid and found that DEs (%) of mAP for cohesive and blunt ends were comparable with those of commercial APs. Therefore, because mAP exhibited excellent efficiency in linearized DNA dephosphorylation and was inactivated by simple heat treatment, further studies may be focused on elucidation of its potential use in molecular biology applications under optimal conditions.

## Conclusions

This study provides a novel AP isolated from metagenomes of ocean-tidal flat sediments. The metagenome-derived AP is expected to show high potential for diverse molecular biology applications including the construction of recombinant plasmids. This was corroborated by its detailed biochemical characterization, which revealed high activity, catalytic efficiency, and thermal instability. In addition, the production of this enzyme with high yield in *E. coli* may be accelerating its applications.

## Methods

### Bacterial strains, plasmids, and chemicals

*E. coli* BL21(DE3) was used as a host strain for overexpression of *mAP* inserted into the pET-21a plasmid (Novagen, Darmstadt, Germany). DH5α cells and pUC19 plasmid (New England Biolabs, Ipswich, MA, USA) were used for plasmid preparation and dephosphorylation assays, respectively. Commercial bacterial AP (BAP, Invitrogen; Carlsbad, CA, USA), Antarctic AP (AAP; New England Biolabs), shrimp AP (SAP; Promega, Madison, WI, USA), calf intestinal AP (CIAP; Roche, Indianapolis, IN, USA), and APex heat-labile AP (HLAP; Epicentre, Madison, WI, USA) were purchased from each vendor. If not stated otherwise, *E. coli* cells were grown in Luria Bertani (LB) medium (5 g/L yeast extract, 10 g/L tryptone, and 5 g/L NaCl) supplemented with appropriate antibiotics. All chemical reagents used were of analytical-laboratory grade.

### Isolation of DNA and fosmid library construction

Metagenomic DNA was isolated from the ocean-flat tidal sediments of the west coast of Korea (Taean, Korea) using a hydroshear machine (GeneMachines, Genomic Instrumentation Services, CA, USA). A metagenomic DNA fosmid library was constructed in *E. coli* EPI300 cells harboring fosmid pCC1FOS using a CopyControl Fosmid Library Production Kit (Epicentre) according to the manufacturer’s protocol. The size of the metagenomic library was estimated to be 600 Mbp, and the average insert size was 30 kb. For functional screening of AP, a pGESSv4 plasmid [19] was electrophoretically transformed into *E. coli* EPI300 cells carrying the metagenomic library. The resulting library cells were grown in LB broth containing ampicillin (50 μg/mL) and chloramphenicol (12.5 μg/mL) at 30°C for 12 h prior to fluorescence-activated cell sorting (FACS). False-positive cells were removed from total library cells using a FACSAria I (BD Bioscience, NJ, USA). A blue laser (488 nm) and a bandpass filter (530/30 nm) were used to analyze enhanced green fluorescent protein (EGFP) fluorescence. Forward and side scatter were used to exclude debris and dead cells. Nonfluorescent cells (10^6^ cells) were collected and subsequently grown in LB broth containing 1 mM *p*-nitrophenyl phosphate (*p*NPP; Sigma Aldrich, St. Louis, MO, USA) at 30°C for 16 h. Next, approximately 200 fluorescent cells were sorted and grown on LB solid medium containing *p*NPP. After incubation at 30°C for 48 h, 47 colonies showed green fluorescence in a fluorescence image analysis. Among 47 colonies, six strongly fluorescent colonies were further examined for fluorescence intensity, representing the presence of the substrate, to confirm cellular phosphatase activity. Finally, a fosmid isolated from one colony displaying phosphatase activity was digested with BamHI and inserted into the pSTV28 plasmid (Takara Bio, Shiga, Japan) to yield a shotgun library in *E. coli* DH10B cells harboring pGESSv4. The shotgun library cells were grown on LB solid medium with *p*NPP, and six different colonies exhibiting fluorescence were analyzed by plasmid purification, followed by enzymatic digestion and DNA sequencing. The six purified plasmids were termed pSTV28-AP1–6.

### Overexpression of *mAP*

The open reading frame (ORF) of putative *mAP* was amplified by polymerase chain reaction (PCR) with the pSTV28-AP6 plasmid as a template using the following primers: 5′-GGGAATTC*CATATG*TCACAATCCTATGCCTGTGC-3′ (italics: NdeI restriction site) and 5′-CCG*CTCGAG***TGAAATCCTCCCTCGAT**GCGGCAACTGCTCTCGG-3′ (italics: XhoI restriction site; bold: factor Xa cleavage site). The PCR product was then inserted into the pET-21a plasmid digested with NdeI and XhoI restriction enzymes, resulting in plasmid pET-mAP harboring the C-terminal His_6_ tag. For overproduction of mAP enzyme, *E. coli* BL21(DE3) cells transformed with pET-mAP were grown in LB medium supplemented with 50 μg/mL ampicillin at 37°C until reaching an optical density at 600 nm (OD_600_) of 0.4–0.5. Cells were then induced with 0.5 mM isopropyl β-D-1-thiogalactopyranoside (IPTG). The culture was transferred to 15°C and incubated for 40 h.

### Cellular fractionation

For cytoplasmic and periplasmic protein preparations, we used a PeriPreps Periplasting kit (Epicentre), which uses osmotic shock to disrupt the outer cell membrane, followed by treatment with lysozyme to digest the cell wall. Briefly, the induced cells were harvested by centrifugation at 6,000 × *g* for 20 min at 4°C and resuspended in periplasting buffer (200 mM Tris–HCl [pH 7.5], 20% [w/v] sucrose, 1 mM EDTA, and 30 units/μL Ready-Lys lysozyme). After incubation on ice for 5 min, osmotic shock was accomplished by quickly adding an equal volume of cold water. This suspension was incubated on ice for 5 min and centrifuged at 12,000 × *g* for 2 min to remove the remaining cells. The supernatant was recovered as the soluble periplasmic protein fraction. The remaining insoluble fraction was resuspended in PeriPreps lysis buffer (10 mM Tris–HCl [pH 7.5], 50 mM KCl, 1 mM EDTA, and 0.1% [w/v] deoxycholate) with complete protease inhibitors cocktail (Roche) and incubated at room temperature for 5 min. Cell debris was removed by centrifugation, and the supernatant containing the cytoplasmic cellular protein fraction was transferred to a clean tube.

### Purification of recombinant mAP

To purify the mAP enzyme, the periplasmic fraction was directly loaded on a HisTrap HP column (GE Healthcare Life Sciences) equilibrated with buffer A (25 mM Tris–HCl [pH 7.6], 1 mM phenylmethanesulfonyl fluoride (PMSF), 25 mM NaCl, 5 mM imidazole, and 1 mM CaCl_2_) using an automated chromatography system (ÄKTA FPLC, GE Healthcare Life Sciences, PA, USA). The enzyme was purified according to the manufacturer’s protocol for gradient elution of His-tagged protein. Protein fractions eluted with buffer B (25 mM Tris–HCl [pH 7.6], 25 mM NaCl, 500 mM imidazole, and 1 mM CaCl_2_) were desalted using Vivaspin centrifugal concentrators (Sartorius, Gottingen, Germany) with buffer C (25 mM Tris–HCl [pH 7.6], 1 mM CaCl_2_, and 10% glycerol). The purity of eluted protein was analyzed by sodium dodecyl sulfate-polyacrylamide gel electrophoresis (SDS-PAGE) on 12% gels, and the protein concentration was determined using a protein assay kit (Bio-Rad, Hercules, CA, USA) with bovine serum albumin as the standard.

### AP activity assay

The standard assay for AP activity was carried out at 37°C for 5 min using 0.5 mM *p*NPP as the substrate in 50 mM DEA buffer (pH 9.0) containing 0.5 mM CaCl_2_, unless indicated otherwise. The release of *p*-nitrophenol (*p*NP) in the reaction mixture (1 mL) was monitored with a multilabel microplate reader (VICTOR X5, Perkin Elmer, MA, USA) at 405 nm over the linear period. One unit of enzyme activity is defined as the amount of enzyme required to release 1 μmol of *p*NP from *p*NPP in 1 min at 37°C. Each value is the mean of at least three assays.

### Molecular mass determination by size-exclusion chromatography

Size exclusion chromatography was performed using a Superose 6 HR column (GE Healthcare Life Sciences) equilibrated with an equilibration buffer (25 mM Tris–HCl [pH 7.6], 150 mM NaCl, and 1 mM CaCl_2_). Separation was carried out isocratically at a flow rate of 0.5 mL/min at 25°C. To estimate the molecular mass, a gel filtration calibration kit (GE Healthcare Life Sciences) was used for molecular mass standards: ferritin, 440 kDa; aldolase, 158 kDa; albumin, 67 kDa; ovalbumin, 43 kDa; chymotrypsinogen A, 25 kDa; and RNase A, 14 kDa.

### N-terminal sequencing

N-terminal sequencing was performed on purified precursor and mature mAP enzymes, which were electrophoresed on 12% SDS-PAGE gels and transferred to polyvinylamine difluoride (PVDF) membranes. The first 20 amino acids of the N-terminal sequences of both purified enzymes were determined by the Edman degradation method with an Applied Biosystems Precise Sequencer (Applied Biosystems, NY, USA) at the Korea Basic Science Institute (Daejeon, Korea).

### Temperature and pH optima and thermal stability

The apparent optimum temperature was determined by running the standard assay at temperatures ranging from 20°C to 80°C in 50 mM DEA buffer (pH 9.0). The apparent optimum pH of the enzyme was determined by running the standard assay using 50 mM Tris–HCl buffer or 50 mM DEA buffer for the pH ranges 7.0–8.5 and 7.5–10.5, respectively. The thermostability of purified mAP enzyme or BAP (Invitrogen) was determined by incubation for 15 min at temperatures ranging from 20°C to 80°C and from 20°C to 100°C, respectively. Samples were taken at different time intervals, and the residual activity was determined by the standard assay.

### Substrate specificity and divalent cation effects

To examine the substrate specificity of mAP, the following substrates were used at a final concentration of 0.5 mM: *p*NPP, D-glucose 1-phosphate, *O*-phosphoethanolamine, phosphoenolpyruvate, phytic acid, paraoxon, and methyl parathion in 50 mM DEA buffer (pH 9.0). In addition, adenosine monophosphate (AMP), adenosine diphosphate (ADP), adenosine triphosphate (ATP), deoxyadenosine triphosphate (dATP), deoxycytidine triphosphate (dCTP), deoxyguanosine triphosphate (dGTP), and deoxythymidine triphosphate (dTTP) were individually used as substrates in 50 mM Tris–HCl buffer (pH 8.5).

The effects of divalent cations were also examined for their influence on enzyme activity. After treating the purified mAP with 5 mM ethylenediaminetetraacetic acid (EDTA), several divalent cations (NiCl_2_•6H_2_O, CoCl_2_•6H_2_O, CaCl_2_, CdCl_2_, CuSO_4_•5H_2_O, MgCl_2_, or ZnSO_4_•5H_2_O) were individually added to the mAP enzyme solution. This enzyme solution was incubated for 15 min on ice, and the recovered activity was measured by the standard assay.

### DNA dephosphorylation assay

The pUC19 plasmid was digested with HindIII, PstI, or SmaI to generate 3′-protruding cohesive, 5′-protruding cohesive, or blunt-ended linearized plasmids, respectively. Linearized plasmids (1.0 μg) were incubated with 1 U of each AP in dephosphorylation buffer for 2 h at 37°C, and the reaction was stopped by incubation at 65°C for 15 min. After linearization and dephosphorylation, DNA fragments were purified using a DNA purification kit (Qiagen). The dephosphorylation buffer for mAP was 50 mM Tris–HCl (pH 8.5) containing 1 mM CaCl_2_, while the buffers for commercial AAP, SAP, CIAP, and HLAP were provided by each manufacturer. A total of 0.1 μg of AP-treated linear plasmid DNA was ligated using 1 U of T4 DNA ligase (New England Biolabs) and incubated overnight at 16°C. Lastly, 10 ng of the ligation mixture was transformed into competent *E. coli* DH5α cells using the standard CaCl_2_ transformation protocol, and the cells were grown on LB solid medium supplemented with 50 μg/mL ampicillin. The control experiment was conducted using sterile water instead of APs in the dephosphorylation step. The percent dephosphorylation efficiency (DE (%)) was calculated from the following equation described in the previous study [30]:

$$ \mathrm{D}\mathrm{E}\ \left(\%\right)=\left(1-\frac{\mathrm{CFU}/\mathrm{m}{\mathrm{L}}_{\mathrm{AP}}}{\mathrm{CFU}/\mathrm{m}{\mathrm{L}}_{\mathrm{control}}}\right) $$

where colony forming unit (CFU)/mL is the number of CFU per milliliter of transformed cells and CFU/mL_AP_ and CFU/mL_control_ are estimated with dephosphorylated plasmids and nondephosphorylated plasmids, respectively.

### Sequence analysis

Automated DNA sequencing of small inserted plasmids was carried out using an ABI377 instrument (Applied Biosystems, Foster City, CA, USA) and dye terminator chemistry according to the manufacturer’s protocol. We used 454 sequencing technology to establish large fosmid sequences. The amino acid sequence of the putative mAP was aligned with those of the homologous proteins using ClustalW (DNASTAR, WI, USA). Phylogenetic analysis was conducted using the MEGA 5.2 program [38].

### Nucleotide sequence accession number

The complete nucleotide sequence of mAP was deposited in GenBank under accession number GQ250428.
